# Adjusting natriuretic peptide decision limits for BMI for a more personalized diagnosis of heart failure

**DOI:** 10.1515/almed-2025-0020

**Published:** 2025-03-24

**Authors:** Damien Gruson, Sergio Bernardini, Marco Perrone

**Affiliations:** Department of Laboratory Medicine, Cliniques Universitaires St-Luc and Université Catholique de Louvain, Brussels, Belgium; Diabète et Nutrition, Pôle de Recherche en Endocrinologie, Institut de Recherche Expérimentale et Clinique, Cliniques Universitaires St-Luc and Université Catholique de Louvain, Brussels, Belgium; Department of Experimental Medicine, University of Rome Tor Vergata, Rome, Italy; Department of Clinical Sciences and Translational Medicine, University of Rome Tor Vergata, Rome, Italy

**Keywords:** heart failures, biomarkers, BMI, obesity, decision

To the Editor,

Heart failure (HF) continues to pose significant challenges globally, with natriuretic peptides playing pivotal roles in its diagnosis and management [1]. These biomarkers are crucial in distinguishing cardiac from non-cardiac causes of dyspnea, for the risk evaluation of HF patients and in monitoring therapeutic efficacy [1], [2].

The increasing prevalence of obesity profoundly affects health systems, especially in cardiovascular disease management. An inverse relationship between body mass index (BMI) and natriuretic peptides levels complicates the biomarker’s use in obese patients, who typically exhibit lower levels, potentially delaying heart failure diagnosis and treatment, and leading to worsened outcomes [3], [4]. This decrease involves several mechanisms. First, adipose tissue, abundant in obesity, expresses natriuretic peptide clearance receptors (NPR-C) which facilitate rapid peptide removal from circulation, significantly reducing their levels [3]. Additionally, there is evidence of reduced myocardial peptide release in obese individuals, possibly due to altered hemodynamic states or adipokine-mediated inhibition of peptide synthesis in cardiac myocytes [4]. Obesity also induces biochemical changes that might influence natriuretic peptide levels. Elevated inflammatory cytokines could disrupt cardiac and renal functions governing peptide production and clearance. Impaired renal function, often accompanying obesity, complicates the renal clearance of natriuretic peptides, influenced by common co-morbidities such as hypertension and diabetes [5]. Moreover, the increased expression of NPR-C in obesity not only enhances clearance but may also engage in hormonal regulation mechanisms, altering physiological balance and contributing to a faster degradation rate of natriuretic peptides [4]. Additionally, genetic factors might influence the synthesis, release, or degradation of natriuretic peptides, contributing to their variability in obese populations [6]. The deficiency observed might also have direct metabolic repercussions, as these peptides are involved in lipid metabolism and influence processes like lipolysis and insulin sensitivity, thus exacerbating metabolic dysregulation in obesity [4].

This complexity underscores the need for more personalized approaches in diagnosing and treating obese patients in clinical settings. Studies by Kozhuharov et al. highlight the need to adjust natriuretic peptides decision limits based on BMI to enhance diagnostic precision for obese patients [7]. These proposed adjustments −3 3 % reduction for a BMI of 30.0–34.9 kg/m^2^ and 50 % for BMI ≥35.0 kg/m^2^ – are designed to make NT-proBNP a more effective biomarker in this population.

Despite scientific support for adjusted cutoffs, integrating them into clinical practice involves overcoming hurdles, including the inertia of established protocols and the need for consensus among healthcare providers. Future research should focus on validating these adjusted cutoffs through rigorous multicenter trials across various populations, including racially and ethnically diverse groups. From a policy standpoint, health authorities should consider revising guidelines to incorporate these new cutoffs, complemented by educational programs for healthcare providers to facilitate a smooth transition.

In conclusion, the rising prevalence of obesity necessitates an adjustment in clinical approaches used to diagnose heart failure, particularly concerning the use of natriuretic peptides. By adopting BMI-adjusted decision limits for this crucial biomarker, the medical community can improve diagnostic accuracy and patient outcomes in heart failure management, ensuring that all patients, regardless of body composition, receive optimal care.
